# Use of AI Histopathology in Breast Cancer Diagnosis

**DOI:** 10.3390/medicina61101878

**Published:** 2025-10-20

**Authors:** Valentin Ivanov, Usman Khalid, Jasmin Gurung, Rosen Dimov, Veselin Chonov, Petar Uchikov, Gancho Kostov, Stefan Ivanov

**Affiliations:** 1 Medical Simulation and Training Center, Medical Faculty, Medical University of Plovdiv, Department of Surgery, University Hospital ‘’Kaspela’’, 4002 Plovdiv, Bulgaria; caspela@abv.bg; 2 Medical Faculty, Medical University of Plovdiv, 4002 Plovdiv, Bulgaria; usmankhalid957@gmail.com (U.K.); jasmingurung12@gmail.com (J.G.); 3 Department of Special Surgery, Medical Faculty, Medical University of Plovdiv, 4001 Plovdiv, Bulgaria; rosendimov68@gmail.com (R.D.); puchikov@yahoo.com (P.U.); sivanov2455@gmail.com (S.I.); 4 Department of General and Clinical Pathology, Medical University of Plovdiv, 4002 Plovdiv, Bulgaria; vesko06@gmail.com

**Keywords:** breast cancer, diagnostics, histology, digital pathology, artificial intelligence

## Abstract

*Background and Objectives*: Breast cancer (BC) is a global health concern for women; the disease contributes to significant morbidity and mortality. A key element in the diagnosis of BC involves the histopathological diagnosis, which determines patient management and therapy. However, BC is a multifaceted disease, limiting access to early diagnosis and, therefore, treatment. Artificial intelligence (AI) is transforming diagnostics in the medical field, especially in the detection of BC. Due to the increased availability of digital slides, it has facilitated the effective integration of AI in breast cancer diagnosis. Diagnosis poses a great challenge, even for experienced pathologists, due to the heterogeneity of this malignancy. Analysing microscopic slides by pathologists requires a considerable amount of time. Implementation of AI into routine workflows holds potential to improve diagnostic sensitivity and inter-observer concordance, and to increase efficiency by reducing the review time, thereby helping to alleviate the burden of diagnosing BC. Previous studies mainly address imaging modalities or oncology broadly, while a few specifically concentrates on the histopathological aspect of breast cancer. This review aims to explore the novel synthesis of AI advancements in digital pathology, including tumour classification, grading, lymph node staging, and biomarker evaluation, and discuss their potential incorporation into clinical workflows. We will also discuss the current barriers and prospects for future advancements. *Materials and Methods*: A literature search was conducted in PubMed and Google Scholar using the mentioned keywords. Articles published in English until July 2025 were reviewed and synthesised narratively. *Results*: Recent studies demonstrate that AI models such as convolutional neural networks (CNNs), YOLO, and RetinaNet achieve high accuracy in tumour detection, histological grading, lymph node metastasis localisation, and biomarker analysis. The reported performance values range from 75% to over 95% accuracy across various tasks, with gains in diagnostic sensitivity and inter-observer concordance, and reduced review time in assisted workflows. However, certain limitations, such as data variability, external validation in clinical practice, and ethical concerns, restrict the growth and optimal performance of AI and its clinical applicability. *Conclusions*: The future for AI looks promising, as it is rapidly evolving. By analysing evidence across multiple domains, this review evaluates both opportunities and persisting barriers, offering practical overviews for future clinical transition. AI cannot replace pathologists; however, it has the capabilities to enhance diagnostic precision, efficiency, and ultimately patient outcomes. It is only a matter of time before AI is adopted into healthcare.

## 1. Introduction

Breast cancer (BC) continues to exert a devastating toll worldwide, claiming the lives of millions of women each year. In 2020, BC was the most common cancer among women, resulting in 24.5% of all cancer cases and 15.5% of cancer deaths [1]. According to the most recent estimate from Global Cancer Observatory (GLOBOCAN) 2022, BC remains the most frequently diagnosed worldwide, ranking second and fourth in incidence and mortality, respectively. It was responsible for 670,000 deaths, making it the leading cause of cancer-related mortality in women [2]. It is essential to understand the development of breast tumours to reduce morbidity and mortality rates in women. However, breast cancer is a complex disease with various categories, each displaying unique biological, clinical, and treatment behaviours [3]. In daily practice, doctors utilise multiple imaging modalities, including radiological images, routine ultrasound, mammography, and Magnetic Resonance Imaging (MRI) before reaching a final conclusion [4,5]. Mammography has significantly reduced BC-related mortality by approximately 30%; however, the accuracy of mammograms may differ due to heterogeneity factors like breast composition and tumour characteristics, which can lead to potential misdiagnosis [6]. Sometimes, it may give rise to certain issues like overdiagnosis [7], overtreatment, and false positives, contributing to unnecessary expenses, biopsies [8], and psychological distress [9]. However, these non-invasive methods may not fully capture the unique characteristics of breast cancers. Consequently, breast pathology remains the gold standard for diagnosis. This involves collecting tissue samples, mounting glass slides, and applying various staining techniques to visualise morphological and immunophenotypic features. Modern treatment strategies involve prognostic and diagnostic stratification of patients to enable tailored therapy. The classification depends on expert assessment of pathology slides [10].

Classical pathological assessment is predominantly dependent on the expertise of the pathologists to analyse microscopic characteristics from tissue biopsies and surgical resections. However, they face various challenges, such as substantial workloads, interobserver variability, and the complexity of the microscopic images, which necessitate the need for novel approaches. The development of digital pathology, driven by whole slide imaging (WSI), has revolutionised the histopathological diagnosis of BC [11,12].

In the past three years, artificial intelligence (AI) applications in pathology have expanded rapidly due to increased computational power, and the curation of large datasets has propelled machine learning in numerous computer vision tasks, including the healthcare domain. Digital pathology was initially developed for capturing and managing high-magnification images of pathology slides, but it is currently expanding into clinical workflows. In recent years, the two domains between computer scientists and pathologists have merged, accelerating the use of artificial intelligence (AI) in digital slide analysis to optimise diagnostics and prognostics, ultimately allowing us to make accurate predictions and enhance the efficiency of the diagnostic workflow [13,14,15]. Therefore, it highlights the need for urgent re-evaluation in the role of AI in breast cancer diagnostics. There is a global shift towards computer-assisted decision-making in various clinical domains of Medicine, and breast cancer diagnostics is no exception. Novel algorithms applying dynamic time warping and growing region seed methods displays potential in mammography through automated analysis of morphological features and tissue asymmetry. The aim of this review is to summarise recent advances in AI applications in BC histopathology, focusing on four key areas: tumour classification, grading, lymph node staging, and biomarker analysis. Differing from earlier reviews which mainly focuses on imaging modalities or oncology more broadly, this review provides a focused synthesis of AI in breast cancer histopathology, highlighting both technical and real-world challenges such as clinical translation, reproducibility, and multimodal data fusion. This unique emphasis offers the international scientific community an updated perspective on the significance of AI in advance pathology-driven diagnostics in BC [16].

## 2. Materials and Methods

This study is a narrative review of the implications of AI in the diagnosis of breast cancer. We searched Google Scholar and PubMed databases for articles published until July 2025 utilising a combination of search terms such as: “breast cancer”, “diagnostics”, “histology”, “digital pathology”, and “artificial intelligence”. Only articles published in English were considered.

Inclusion Criteria:

1. Original research articles, systematic reviews, or meta-analyses related to BC in the context of AI, machine learning, or digital pathology.
2. Studies published in English.
3. Articles reporting clear methods and results relevant to diagnostic or prognostic applications.

Exclusion Criteria:

1. Non-English publications.
2. The full text was not accessible.
3. Conference abstracts, preprints, and case reports.

**Figure 1:**
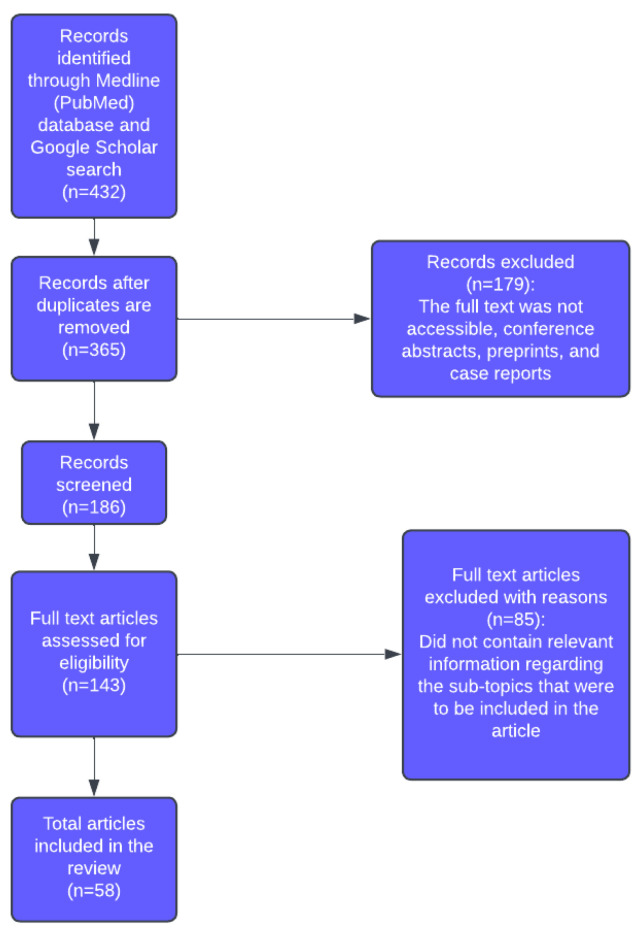
The selection process is summarised below.

Figure 1 highlights our materials and methods in a detailed workflow, showcasing the steps taken to obtain our data.

## 3. Results

### 3.1. Histology

Histopathology is a fundamental pillar in tumour diagnosis, allowing pathologists to accurately divide the tissue sections into benign and malignant features and to gain further insights such as tumour grade and aggressiveness. Immunohistochemical (IHC) staining for assessment of key biomarkers, including oestrogen receptor (ER) and progesterone receptor (PR) expression, continues to tailor the treatment plan for patients [17]. Despite current guidelines, biomarker assessment is relatively limited due to the diversity of tissue sections, and the interpretation of the visibility of biomarkers varies between different individuals [18,19]. Many studies show inconsistencies between biomarker analysis, consequently rendering the discovery of new biomarkers and their utilisation in patient care [20].

Innovations in digital pathology and computational image analysis have begun to alleviate the initial limitations that challenge its use in clinical medicine. The availability of high-quality digital images now enables more consistent analysis of morphological and biological features, reducing inter-observer variability and improving reproducibility [21]. With the application of AI, histological assessment shows potential to enhance both biological evaluation and morphological pattern recognition, offering results that are more standardised and reproducible compared to manual slide examination alone. While traditional histology remains central to diagnosis, the integration of AI-based tools is steadily improving precision and reliability in histopathological evaluation, making promising strides [22].

### 3.2. Digital Pathology

Digital pathology refers to the translation of histopathological slides into the digital world via WSI scanners and is stored in an image management system (IMS). It consists of the acquisition, management, and interpretation of the individual slides. Pathologists can access WSIs utilising specialised software at multiple magnification scales [12,23,24]. To ensure safe and effective use of digital methods, a clinical validation of BC diagnosis from digital slides was performed based on the Royal College of Pathologists’ guidelines. Three pathologists received a digital pathology training course, and the initial training consisted of 20 challenging test cases. Clinical concordance between glass and digital slide was obtained in 98.8% of cases, with 1.2% demonstrating clinical significance. Structured training ensures pathologists gain confidence and competence before utilising digital platforms [25]. Williams et al. conducted a study to assess the safety and benefits of digital pathology systems for preoperative tissue diagnosis of screen-detected BC lesions. Digital versus glass slide diagnosis achieved a very high clinical concordance of 96% (216/225) and 99.6% (249/250) in experimental data from two departments, whereas validation studies utilising the Royal College of Pathologists protocol received 99.4% (180/181) and 99.0% (887/896). The intraobserver variability demonstrated a kappa statistic of 0.80, exhibiting excellent agreement [26].

**Figure 2:**
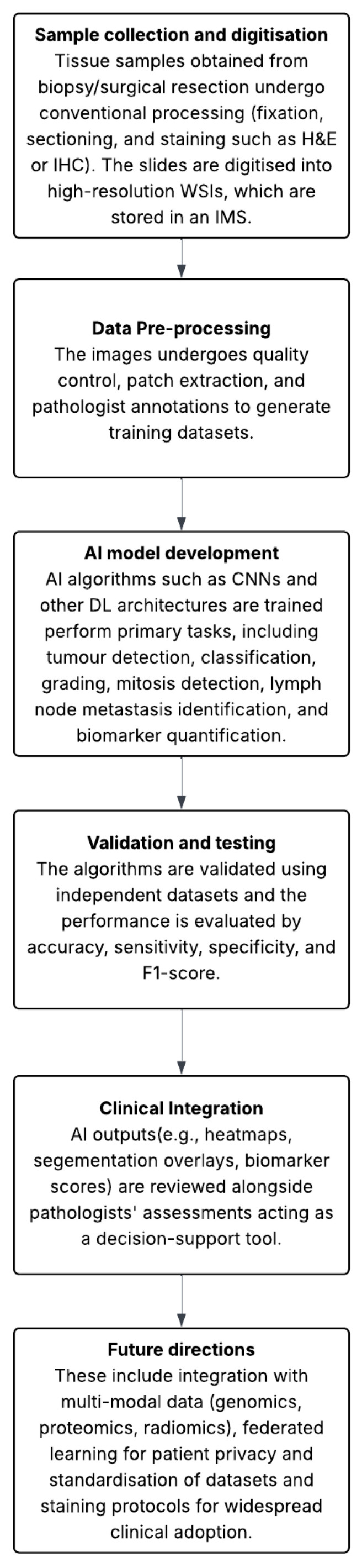
Illustrates the schematic workflow of AI in histopathology, including sample collection and digitisation, data pre-processing, AI model development, validation and testing, clinical integration, and future directions.

In this research, Zhao et al. developed a single-cell morphological and topological profiling (sc-MTOP) framework. They were able to extract specific features from 410 million cells from WSIs of 637 BC samples to group them into four BC ecotypes. This, coupled with multiomics, revealed that tumours with locally clustered inflammatory cells respond better to immunotherapy in triple-negative breast cancers. Intratumour diversity also demonstrates activity of the cell cycle pathway activation. Additionally, it also highlights the degree to which CDK inhibitors work in hormone receptor-positive cases [27]. Figure 2 highlights the diagnostic workflow with clinical integration for AI within histopathology based breast cancer diagnosis.

### 3.3. Artificial Intelligence in Breast Cancer Diagnosis

The accelerated expansion of affordable computer power, along with the availability of cloud computing resources, enables increased availability of digital data and inexpensive storage of this information. Artificial intelligence (AI) is integrated into many everyday devices such as computers, mobile phones, cars, and smartwatches, where its applications vary from robotics, management of junk emails, and voice processing. AI consists of an array of techniques where the machines are programmed to stimulate aspects of human cognition, coupled with the capacity to learn [28].

Deep learning (DL) algorithms built upon computer-aided detection/diagnosis (CADe/CADx) represent more recent advancements. It utilises one or multiple neural network (NN) architectures, using neural architectures inspired by biological information processing, resulting in more accurate data-driven decisions. Compared to traditional machine learning techniques, which manually select features, DL implements a unique process known as backpropagation, enabling automated learning and optimisation [29]. Various branches of DL exist; one of the most commonly used models in pathology image analysis is Convolutional Neural Networks (CNNs), which applies previously trained filters onto input images, allowing extraction of complex characteristics resulting in precise detection of patterns [30].

Cruz-Roa et al. developed a powerful method based on CNNs called Histopathology Image Analysis (HASHI), which processes images using probability gradients and quasi-Monte Carlo sampling to determine whether a BC whole slide image (WSI) contains invasive ductal carcinoma. The model was trained on three different data cohorts consisting of nearly 500 cases and validated on 195 studies from The Cancer Genome Atlas, obtaining a pixel-level F1 score of 76%. HASHI assesses 2000 samples in just one minutes, compared to 6 million samples in 24 hours, highlighting its efficiency [31]. Alternatively, Han et al. developed a classifier capable of eight multi-classifications using the BreaKHis dataset, obtaining an average accuracy of 93.2% on a large-scale dataset [32].

While the primary scope of this review is histopathology, there are several notable AI developments in breast imaging (e.g., ultrasound, mammography) that demonstrate parallel progress. Contemporary breast ultrasound Computer-Aided Diagnosis (CAD) systems require a predetermined region of interest (ROI) to be defined by a radiologist. This process is slow and prone to variability among human experts. Yap et al. developed a DL tool for BC mass diagnosis utilising Faster Region-Based CNN (FRBCNN), achieving an average recall rate of 0.9236, a precision rate of 0.9408, an F1-score of 0.9321, and a false alarm rate of 0.0621. The results were further refined when using red, green, and blue images, obtaining a recall rate of 0.9572, a precision rate of 0.9020, an F1-score of 0.9288, and a false alarm rate decreased to 0.1111 [32]. Another framework employing Faster Region-Based CNN (Faster RCNN) for the detection of BC masses in Full-Digital Mammograms (FFDM) achieved a True Positive Rate (TPR) of 0.93 and a False Positive per Image (FPI) of 0.78 on a public dataset. On another public dataset known as INbreast (Siemens scanner), the framework demonstrated higher performance of TPR of 0.85 at 1.0 FPI for benign masses and 0.99 at 1.17 FPI for malignant masses [33].

RetinaNet is a DL model consisting of a backbone and two smaller subnetworks for object detection. This model leverages Focal Loss, mitigating the imbalance between positive and negative samples to strengthen performance. The model accomplished the highest performance to date with very low mean False Positive Indications per image (mFPI) of 0.45–0.47 and partial area under the curve (AUC) of 0.93 across multiple datasets for the detection of benign and malignant masses. In addition, it was capable of detecting various manifestations such as: architectural masses, distortion, and microcalcifications [34]. Unlike other models, a novel model known as You Only Look Once (YOLO) is a DL model possessing the ability to handle detection and classification within one unified framework. In this CAD system, 600 mammograms from the Digital Database for Screening Mammography (DDSM) and 2400 augmented mammograms were utilised to build the system. This system gained an overall accuracy of 99.7% and was able to further differentiate benign and malignant lesions with an overall accuracy of 97% across five-field cross-validation tests. While these results are impressive, they must be interpreted with caution, as the near-perfect results may reflect overfitting to specific datasets, risk of data leakage, or lack of external validation [34]. Su et al. developed a model integrating both YOLO and Local-Global (LOGO) for effective mass detection and segmentation simultaneously. The results were evaluated on the Curated Breast Imaging subset (CBIS) of DDSM and INBreast mammography datasets, exceeding prior works with a TPR of 95.7%, a mean average precision of 65.0%, F1-score for mass segmentation with 74.5%, and Intersection over Union (IoU) with 64.0% [35]. These radiology studies are included to provide context on how different AI algorithms perform across modalities; however, the primary focus of this review is the application of AI to histopathological analysis. Table 1 provides a focus of summary for the various AI models discussed in the review.

This table compares key AI models in breast cancer diagnosis. Cruz-Roa et al. demonstrate that CNNs can accurately and efficiently detect invasive breast cancer regions, while Han et al. can further classify the histological images with high accuracy. Yap et al. displayed a novel algorithm, FRBCNN, capable of lesion localisation in ultrasound images, and Ueda et al. presented strong performance of RetinaNet across different datasets. Su et al. combined YOLO and LOGO models for effective detection of breast mass detection and segmentation simultaneously. Despite the various benefits of AI, clinical translation is restrained due to annotation workload, reproducibility across different centres, reliability on curated datasets, therefore limiting the real-world application.

### 3.4. Role of AI in Diagnosing Lymph Node Metastasis in Breast Cancer

BC staging is crucial in the clinical management of patients. A key aspect of this includes neoplastic dissemination via histopathological assessment of sentinel axillary lymph nodes (SLNs). The Cancer Metastases in Lymph Nodes Challenge 2016 (CAMELYON16) was the first major competition utilising WSIs. In this cross-sectional analysis, seven DL algorithms displayed superior results in comparison to a panel of 11 pathologists, with an AUC of 0.994 for the best algorithm and 0.884 for the best pathologist in a time-constrained diagnostic setting [38]. Pathologists manually assess the metastasis by counting the number of tumour cells present and/or measuring the size to stratify the type of metastasis as macrometastasis, micrometastasis, or isolated tumour cells to employ the best treatment plan for patients. A custom-made algorithm analysed 135 BC patient samples who underwent SLN biopsy, showing 100% sensitivity (no false negatives), therefore decreasing pathologists’ workload by 58.2% [39]. To assess the effect of digital assistance, six pathologists reviewed 70 digital slides with and without the DL algorithm’s support. The results were promising, increasing sensitivity from 83% to 91% and the review time for micrometastasis decreased from 116 s to 61 s. Moreover, pathologists reported interpretation of micrometastasis far easier with assistance (*p* = 0.0005) [40]. Similarly, Liu et al. assessed the addition and clinical implementation of a lymph node assistant algorithm for metastatic BC in SLN biopsies. This algorithm demonstrated an AUC of 99% and a tumour-level sensitivity of 91% with no alterations from histological artefacts such as poor staining, air bubbles, and over fixation [41].

An essential task in BC staging is identifying metastasis. In most cases, pathologists analyse tumour characteristics and metastasis areas in WSIs; however, this process is time-consuming and vulnerable to mistakes. Iqbal et al. employed patch-based algorithms to isolate small sections from larger WSIs to pinpoint tumour locations [42]. The overlapping regions in the images may also lead to computational redundancy. To overcome this, Wang et al. developed an innovative two-stream network to identify tumour metastasis by simultaneously analysing high- and low-magnification patches. It was assessed on a dataset referred to as Camelyon16. This method displayed a free-response receiver operating characteristic (FROC) score of 0.871 with a rapid inference time compared to existing methods. Additionally, it demonstrated a FROC score of 0.88 with a high magnification network [43]. A different study addressing this issue presented a “metastasis AI” detection app with the Visiopharm Integrator System (VIS) metastasis AI algorithm. It was able to detect all metastases in both sentinel and nonsentinel lymph node samples with 100% sensitivity and negative predictive value (NPV). These results are promising and could be integrated into the routine digital pathology workflow to increase efficiency [44].

### 3.5. Role of AI in the Histological Grading of Breast Cancer

In 2018, Fondón et al. developed a CAD tool utilising support vector machine classifiers with quadratic kernels to classify the samples into: normal, benign, in situ, and invasive. Upon initial five-fold cross-validation with 120 images, it was tested on an external set of 30 different images, including some with artefacts. It achieved accuracy levels of 75.8%, 75%, and 61.11%, respectively. Hence, it can aid in BC diagnosis, preventing avoidable deaths [45]. In BC development, the evolution from the pre-invasive state of ductal carcinoma in situ (DCIS) to invasive ductal carcinoma (IDC) is vital [46]. Myoepithelial cells help differentiate benign and proliferative breast disease from invasive tumours in final needle aspiration cytology smears.

Additionally, they are key in the diagnosis of DCIS and IDC [47]. Assessing subtle variations in 11,661 myoepithelial cell nuclei, Yamamoto et al. developed a model to classify different histological types of intraductal proliferative lesions. The myoepithelial nuclei cells displayed a unique flattened shape, detectable by computational methods, and were able to categorise four histological subtypes with 90.9% accuracy. Not only does this potentially enhance diagnostic accuracy, but it can also help aid our understanding of BC pathology [48].

Current pathologists categorise the aggressiveness by grading, which entails assessment of the morphological features of the samples, for example, tumour and epithelial cells for tubular formations and mitotic cells for mitotic count. Not only is this a laborious task, but there is also variability in the observations. Apart from tumour identification, recent advancements in AI make it possible to analyse aggressiveness via mitosis. Veta et al. presented the results from the Assessment of Mitosis Detection Algorithms 2013 (AMIDA13). The leading performer, labelled IDSIA, demonstrated an overall F-1 score of 0.61 against the consensus of pathologists. On the other hand, individual pathologists obtained an overall F-1 score of >0.75. Their success was attributed to a 10-layer deep CNN algorithm [49]. In 2016, they launched another challenge, termed Tumour Proliferation Assessment Challenge 2016 (TUPAC16), evaluating mitotic scores on WSIs. The most successful method obtained a Cohen’s kappa score of 0.56 with pathologists’ slide-level score and an F-1 score of 0.65 on cell-level mitosis detection [50]. The moderate F1-scores (0.65–0.75) highlight the ongoing challenge of automated mitosis detection. This represents biological variability, inconsistencies in annotation, and poor generalisability across heterogeneous datasets, emphasising the need for enhanced methodological approaches [49,50].

In one study, Pantanowitz et al. concluded that pathologist end-users were far more accurate and efficient when supported by AI in evaluating mitotic figures in digital slides of invasive BC. It was shown that 87.5% of readers (21/24) were able to detect more mitosis, and 54.2% (13/24) were able to reduce false positives with AI assistance. Overall, the time spent decreased by 27.8% [51].

Tubular formation and nuclear grading are emerging fields of interest in the histological grading of BC. In 2016, Romo-Bucheli et al. engineered a CNN DL classifier capable of automated detection of tubule nuclei from WSI, thereafter predicting Oncotype DX risk categories, acquiring a correlation AUC of 0.76 [52]. Stemming from the adoption of more state-of-the-art and contemporary equipment in pathology labs, AI can be utilised for automatic nuclei segmentation beyond tubule formation, gaining an independent prognostic value of 0.032 [52].

#### Comparative Perspectives on AI Architectures

Different AI architectures have been adopted into histopathological image analysis, each offering unique advantages and limitations. CNNs are the most common, allowing identification of DCIS from IDC [46,47,48] and detection of mitotic figures with good accuracy [49,50]. These classifiers also allow for automated assessment of morphological features, including tubule formation and nuclear grading, providing a prognostic classification as well [52,53]. CNNs’ greatest attribute lies in their ability to identify local features at cellular and subcellular levels. However, they are limited when assessing global tissue context across WSIs, subsequently decreasing performance in heterogeneous datasets [50,51].

To overcome these challenges, alternative algorithms such as support vector machines (SVM) have been explored [45], which demonstrate high performance on small datasets, but often underperform in comparison to DL applied to complex histopathological tasks. Meanwhile, the recent advances in computational capacity have led to deeper CNN models [49,52], displaying improved generalisation and reduced inter-observer variability, but they are highly dependent on high-quality, large datasets [42,43,50,52,53], revealing a shift towards models utilising precise local features with wider tissue-level insights, stressing the need for hybrid and scalable AI models in BC histopathology.

### 3.6. Molecular Pathology

With the advent of new methods, digital image analysis (DIA) can aid in quantitative biomarker evaluation. According to gene expression patterns, BC subtypes are categorised into ‘Luminal A’, ‘Luminal B’, ‘human epidermal growth factor receptor 2 (HER2)-enriched’ and ‘Basal-like’. However, gene expression assays are not readily available; therefore, IHC stains can be used as a replacement. DIA, constructed upon the VIS, exhibited superior sensitivity and specificity compared to manual scoring regarding luminal B, BC subtype, thus decreasing the time for pathologists due to automation of multiple steps [54]. Virtual Double Staining (VDS) involves the fusion of Ki-67 positive and negative tumour cells and cytokeratin (CK) stained images for breast carcinoma classification. Both VDS and manual counting demonstrated high agreement with correlation coefficients greater than 0.97. This method yielded better results in neighbouring slides (>85%), although it was not as efficient in non-neighbouring slides [55]. The application of VDS is also suitable for the detection of ER proteins. In this study, Andersen et al.’s image analysis system demonstrated perfect agreement with human scoring for ER status with 1% cut-off. Using the histoscore method, a very high correlation was also visualised between VDS and visual assessment (R = 0.950) [56].

Beyond current histopathology applications, AI has the potential to integrate with other data modalities such as genomics, proteomics, and radiomics. Radiogenomics bridges imaging and molecular profiles by combining radiomic features with gene expression for precision oncology [57]. The combination of different modalities will allow for far more accurate detection, along with subtyping, therapy, and prognostic evaluation [58]. Previous multimodal studies have shown great promise, illustrating the extent of our progress towards comprehensive precision oncology in BC.

## 4. Discussion

The application of AI is revolutionising diagnostics in BC; however, it is still in its infancy. In this section, we discuss the limitations and challenges AI faces.

For optimal model performance, high-quality data is essential. Multiple algorithms have displayed poor performance because of poorly stained slides and insufficiently annotated datasets [31,53]. Algorithms were only trained for the detection of masses, not classification into benign and malignant, which is crucial in diagnosis [33,41]. Certain models lack clinical relevance, as the dataset was not validated in clinical settings. Moreover, they may produce false positives, potentially leading to adverse consequences. The data consist of numerous cancer cases that are not routinely observed by pathologists [37,38]. Others lack clinical validation, as the training environment does not fully mimic the routine scenario in hospitals, and as each pathologist only analysed one stained slide per patient; however, in hospitals, they meticulously examine multiple slides at different levels for diagnosis. Additionally, they may also perform supplementary methods like IHC staining when cases are difficult to diagnose. The training environment is unrealistic, demanding pathologists to review 129 slides in two hours, which is not representative of everyday practice [38,40]. More cases should be incorporated to increase exposure to rare events, thus increasing reliability and generalisability, as some systems are unable to pinpoint the exact location or tumour–lymph node measurements [40,41]. While the concept of AI fully taking over seems appealing, it cannot replace doctors, as it requires expert guidance along with human oversight [42]. Older methods such as SVM-based CAD tools provide proof-of-concept; however, these models have been surpassed by modern architectures (e.g., CNNs), which provides improved scalability and robustness [45].

### 4.1. Ethical, Legal, and Privacy Issues

Adoption of AI raises important ethical, legal, and privacy considerations. The training dataset consists of sensitive histopathology and genomic information; therefore, strict compliance with data protection standards is required. A large amount of data is required to build robust models [42]. Another challenge is algorithmic bias, as it is built upon homogenous datasets; it may underperform in unrepresented groups, exacerbating health inequalities [31,37,38]. Consequently, integrating explainable AI frameworks and systematic bias auditing is crucial before it is utilised by clinicians and patients [40,44].

### 4.2. Standardisation and Interoperability

A further challenge is the lack of standardisation across datasets and interoperability. The reproducibility across multiple institutions is limited, as the current models are designed upon heterogeneous datasets, which include inconsistent staining protocols [31,33]. External validation is essential before widespread adoption can be considered [38,40,44,54]. Establishing standardised frameworks is important to achieve interoperability and smooth clinical translation.

### 4.3. Research Gaps and Future Directions

Despite significant progress, important research gaps remain. In the future, algorithms can couple fully convolutional networks with graphics processing units to utilise AI to its full potential [31]. Integration with 3D imaging modalities and optimisation of data according to different countries may enhance the applicability [33,37]. Similarly, the monitors should be standardised for future studies to reduce inconsistencies and bias [51]. Prospective studies need to be carried out before deploying into clinics to establish causality and any confounding factors present [38,40,41,44]. Ultimately, AI cannot function autonomously. It is a supplementary tool; pathologists should not rely solely on AI, and results require validation [44]. The continued development of models with standardisation of frameworks and multimodal integration with genomics, proteomics, and radiomics will be crucial to a safe, effective, and equitable clinical translation.

## 5. Conclusions

With the rapid growth of AI, it provides several advantages to pathologists, including enhanced diagnostics and cuts in time, effort, and expenses. Integration of AI in BC is constantly evolving, and the results have been promising; therefore, they could aid pathologists in their workflow, improving accuracy and efficiency. It holds the power to reshape the diagnostic pathways of BC.

However, adoption of AI into clinical practice is mainly dependent on overcoming key challenges. The training datasets are what hinder the growth of AI, as the quality and performance of AI are dependent on the initial training dataset. Future directions should prioritise integration of histopathology with multimodal data sources, for example, genomics, proteomics, and radiomics, to enhance precision oncology. Incorporation of 3D imaging can also provide a realistic model for tumour morphology. Federated learning represents a progressive approach for the use of diverse, distributed datasets while preserving patient privacy. AI is not a replacement but rather a supplementary tool for pathologists, and it should require human oversight in critical diagnostic decisions. Before AI can be adopted into clinical workflows, it must undergo extensive, large-scale clinical trials for validation. It is just a matter of time before AI can reach its full potential, essentially having a positive impact on patient outcomes, and ultimately, improving the quality of life in women to lessen the global impact.

Overall, the trajectory of AI in histopathology is evolving from algorithm development towards clinical translation, with a focus on standardisation, external validation, and integration into decision-support workflows, highlighting its value as an addition for precision oncology and tailored care towards patients.

## Figures and Tables

**Table 1:** Summary of AI models in breast cancer diagnosis.

Study	Objective	Dataset (Size)	Performance Values	Strengths	Limitations
Cruz-Roa et al. [31]	Detection of invasive breast cancer in WSI using CNN-based adaptive sampling (HASHI)	~500 training cases tested on 195 The Cancer Genome Atlas slides	Dice coefficient: 76%	Highly efficient processing ~2000 WSIs/min	Limited generalisability
Han et al. [32]	Multi-classification of histopathological breast cancer images using DL model	BreaKHis; 7909 images and 8 sub-classes of breast cancer	Accuracy at patient level: 93.2% Accuracy at image level: 93.8%	Multi-classification Good generalisation	Utilises a single dataset (BreaKHis) Does not report other metrics (accuracy, sensitivity, specificity)
Yap et al. [36]	Automated ROI detection and lesion localisation in breast ultrasound images using FRBCNN	Ultrasound dataset A and B	Accuracy recall rate: 0.9236 Precision rate: 0.9408 F1-score: 0.9321 False alarm rate: 0.0621	Leverages novel object detection algorithm (FRBCNN) Uses transfer learning to overcome the limited dataset available	No standard metrics reported Dataset sizes not clear Focuses on localisation not full lesion classification
Ueda et al. [37]	Detecting breast cancers on mammography using DL model	Development dataset: 4636 mammograms Hospital test dataset: 491 images Clinic test dataset: 2821 images	Detected all cancers with a 0.45–0.47 mFPI and partial AUCs of 0.93 in both test datasets	Strong performance across hospital and clinic datasets Used nonmalignant images to help identify normal features Open-source model	Asymmetrical data with more nonmalignant images Difficult detecting malignant lesions in dense breast tissues
Su et al. [35]	Simultaneous breast mass detection and segmentation using YOLO and LOGO models	Two independent test sets	True positive rate: 95.7% Mean average precision: 65% Mass segmentation on CBIS-DDSM dataset with F1-score of 74.5%	Simultaneous detection and segmentation Good performance while maintaining segmentation capabilities	Generalisibility to other datasets not fully shown

## Data Availability

Not applicable.

## Abbreviations

The following abbreviations are used in this manuscript:

BC	Breast Cancer
MRI	Magnetic Resonance Imaging
ER	Estrogen Receptor
PR	Progesterone Receptor
WSI	Whole Slide Image
IMS	Image Management System
NN	Neural Network
CNN	Convolutional Neural Network
ROI	Region of Interest
FRBCNN	Faster Region Based Convolutional Neural Network
FFDM	Full Field Digital Mammography
FPI	False Positive per Image
TPR	True Positive Rate
INbreast	Siemens scanner dataset
mFPI	mean False Positive Indications per image
AUC	Area Under the Curve
YOLO	You Only Look Once
LOGO	Local Global
IoU	Intersection over Union
SLN	Sentinel Lymph Node
DCIS	Ductal Carcinoma In Situ
IDC	Invasive Ductal Carcinoma
VIS	Visiopharm Integrator System
NPV	Negative Predictive Value
DIA	Digital Image Analysis
VDS	Virtual Double Staining
CK	Cytokeratin
CBIS	Curated Breast Imaging subset
CAMELYON16	Cancer Metastases in Lymph Nodes Challenge 2016
IHC	Immunohistochemistry/Immunohistochemical
AMIDA13	Assessment of Mitosis Detection Algorithms 2013
HER2	Human Epidermal Growth Factor Receptor 2
GLOBOCAN	Global Cancer Observatory
SVM	Support Vector Machine
